# The Course of the Bullet in the President’s Wound

**Published:** 1881-08

**Authors:** Edmund Andrews

**Affiliations:** Professor of Clinical Surgery in the Chicago Medical College; No. 6, Sixteenth Street, Chicago


### ARTICLE VI.

### THE COURSE OF THE BULLET IN THE PRESIDENT'S WOUND.

By EDMUND ANDREWS, A.M., M.D., Professor of Clinical Surgery in the Chicago Medical College.

Having recently conversed with Prof. Frank H. Hamilton and others, who have specially investigated President Garfield's wound, I think a few of the facts elicited may interest the profession.

The symptoms are such as make it a matter of doubt what the real internal injuries are. The would-be assassin shot him from behind, but the exact direction of the pistol at the moment of discharge is not clearly known. The attending surgeons agree that the bullet entered the back four inches to the right of the center of the spine, and traversed downward and forward through the mass of spinal muscles, and that it struck the right eleventh rib, and thence passed inside the bony walls of the thorax. The bullet was a conical one of the caliber $\frac{44}{100}$, and weighed 200 grains. The point was truncated, probably to make the cartridge short enough to be used in a short cylinder. This fact, and the additional one, that the charge of powder was light, gives increased probability that in striking the rib its course might be deflected.

The attending surgeons very properly judged that vigorous probing was unsafe. However, Surgeon-General Wales, of the Navy, introduced his little finger, and thought he felt the liver, and that the upper edge of the eleventh rib was notched or injured. Immediately after the shot the President felt pain in the distribution of the right great sciatic nerve, followed not long after by pain felt equally in the distribution of the great sciatics of both limbs. It is not quite easy to account for this fact. A probe is said to have been introduced three and a half inches by another attendant; but every one knows that probes in such cases run into intermuscular spaces with surprising facility, and give very imperfect information. In addition to the pain in the legs, the President felt a sense of heat and deranged sensation in the skin of the right inner part of the thigh, and right side of the scrotum.

The patient vomited repeatedly, but threw up no blood, nor was there any stain of blood in the sputa, alvine discharges, nor in the urine. There was some retention of urine, relieved by the catheter. There was some, but not excessive, shock, followed by a fair, moderate reaction.

The surgeons at Washington believed the liver to be wounded, and judged it probable that the bullet had passed through that organ, and lodged in the anterior wall of the abdomen, or in the groin, possibly wounding intestines on the way. As general peritonitis did not supervene, the fear respecting the intestines subsided, together with the anxiety about hæmorrhage. A spot in the right hypochondrium showed an ecchymosis and tenderness and was thought to be the possible location of the ball.

Now what was the nature of the President's wound? As to the liver having received the bullet, the evidence seems only that the location of the wound rendered it probable. Dr. Wales thought he felt it with his little finger, but liver tissue is not so characteristic to the touch as to secure him against mistake, unless he explored with an energy which would be dangerous and unjustifiable. If the bullet notched the upper edge of the eleventh rib, and thence entered the liver, it must have passed across the lower edge of the posterior part of the right lung, wounding that organ, opening the pleura, and piercing the diaphragm. In such case Dr. Wales would have to pass his finger through the pleural cavity to reach the liver. If this were the course, the patient should have spit blood, and had an escape of air, followed by pleuritis, and at least some pneumonia, neither of which has occurred. If the bullet was deflected downward by the tissues which resisted it, and by striking the eleventh rib, so as to escape the pleura and the lung, such a course would be likely to carry it behind the liver and not into it. I understand Dr. Hamilton that Dr. Wales' examination with the finger gave him the impression that only the upper border of the rib was notched. If this were so, the glancing of the bullet would throw it more forward instead of downward, and the wounding of the lung and the liver would be sure.

As the lung certainly escaped, and as there is not a single clear proof of the liver being touched, it seems necessary to suppose that Dr. Wales, examining, as he doubtless did, briefly, was not able to get an accurate idea of the condition. A splinter of rib driven downward might give him the impression of the upper border of the bone. It is also conceivable that a hasty examination of a somewhat stout man like the President might cause him to mistake the twelfth rib for the eleventh. At any rate there has not been a single decisive symptom of a wound of the liver, so far as I have been able to learn, though plenty of reason to fear its possibility.

Prof. F. D. Weisse, of New York, after experiments and dissections upon the cadaver, authorized the following report in the *N. Y. Herald*:

TO THE MEDICAL PROFESSION AND THE PUBLIC: In view of the fact that I have been reported as having for several days past been engaged in making some investigations bearing upon the probable nature of the pistol shot wound inflicted upon the President, and because the published statements are disjointed and vague, I desire to make this public statement of my theory and observations relative to the case. On the second day after the shooting of the President it seemed possible that the wound did not involve the peritoneum, digestive canal or right kidney, and if so the track and lodgment of the ball were yet unsolved. Studying carefully the official bulletins and the statement of the physicians in attendance it seemed o me that the weight of evidence pointed to an injury of the sacral plexus of nerves. I determined to investigate the subject, hoping to be able to contribute a possible clew to the unravelling of the case. I conceived the theory that by some fortuitous circumstance the ball had been deflected from its course by striking and fracturing the eleventh rib; that it had passed along the anterior surface of the transversalis muscle that forms the interior muscle wall of the outer half of the loin, passing between the transversalis muscle posteriorly and the kidney and ascending portion of the large intestine anteriorly; thence into the iliac fossa (which is the bed of the right groin), between the iliacus internus muscle posteriorly, and the cæcum or commencement of the large intestine anteriorly; thence continuing downward and inward through or behind the psoas magnus muscle to the right side of the pelvis (or lower cavity of abdomen), there inflicting an injury to one of the nerve trunks of the sacral plexus itself, where the ball is now probably lodged. This being the supposed track of the ball it would plow through a mass of fat which exists between the muscle walls behind and the abdominal organs above mentioned in front. This layer of fat in President Garfield (judging from his physique as pictured and described), is probably from an inch and a half to two inches thick. Two nerves cross the supposed track of the ball, lying upon the anterior surface of the transversalis muscle between it and the fat—namely, the ilio-hypogastric and ilio-inguinal branches of the lumbar plexus. These two nerves pass round each side between the muscle planes of the abdominal wall, reaching the groin, where they emerge at the side of the median line at the lowermost point of the belly, distributing nerve films to the skin of that region and to the skin of the scrotum, which areas of skin they endow with sensibility.

The symptoms which seemed to lead to this theory were, first, that the President fell on the receipt of the wound without losing consciousness; second, that he vomited almost immediately after falling; third, that upon being asked immediately after the receipt of the wound if he had any pain, he answered, "Yes, in my right leg and foot;" fourth, the occurrence of tingling and burning of the skin of the legs and feet together with the excruciating pains and cramps of the same; fifth, that upon the cessation of these pains he was left with a feeling of soreness of the muscles and exaggerated sensibility of the feet and legs. To test the theory of the deflection of the ball and its course I investigated the qualities of the weapon that inflicted the wound and made special dissections of the region involved. As regards the pistol used I hope to find some inherent defects in it which might tend to bear out the probability that the ball fired from such a pistol could be deflected; I had even thought that if it did prove a reliable weapon; that the particular cartridge fired might have been a poor one. I obtained a British bull-dog pistol and a box of forty-four caliber cartridges, and upon inquiry was informed that as a weapon it was defective in many particulars, both as to pistol and cartridge; first, the cartridge contains eighteen grains of powder in a very shallow cup, the ball weighing 200 grains; second, that the ball is cut off at the end and not pointed; third, that the shortness of the barrel causes a loss of the effectiveness of the powder at the muzzle in firing; fourth, that as a weapon of precision in firing it is defective in the feeble grip afforded by the small and awkward shape of the handle; also, that the shortness of the barrel tends to elevate the muzzle at the time of the explosion of the cartridge. Upon testing the effectiveness of the weapon I found first upon firing at a one inch board ten feet off that the ball went through the board, but did not have force enough to penetrate a second board eight inches beyond and dropped to the ground; second, firing into the trunk of a suspended cadaver I found whenever the ball struck a flat bone it would lodge in the body; if it did not impinge upon a flat bone of the chest it would at times go straight through, but more often would lodge at an opposite point beneath the skin. Firing into the abdomen it would lodge in the body, owing, probably, to the mobility of the organ. The above observations would seem to show the possibility of a ready deflection in the ball.

The ball that entered the President's body is described as having entered four inches to the right of the median line of the back, fracturing the eleventh rib. The eleventh is the most movable of all the ribs, being free at its anterior end and not steadied, as is the twelfth rib, by a muscle attached to its lower edge. The conditions favorable to reflection by impingement upon and fracture of this rib are—first, the mobility of the rib makes it like a hickory twig fixed at one end only, and it swaying upon impact would expend a good deal of the force of the ball; second, the fracture of the rib would contribute still further to break the force of the ball; third, the external surface of the rib being convex tends to deflect a ball (of all bones of the body the rib probably deflects balls most often); fourth, if the eleventh rib is pushed inward from behind it rises anteriorly, and if a ball struck it, it would tend to turn the ball on its axis and deflect it downward and inward; fifth, the clothing of the President must also be considered as an element contributing to diminishing the penetrating power of the ball before it reached the rib, but I have no data with reference to the clothing. The above conditions at the point of entrance of the ball as impinging upon and fracturing the movable eleventh rib seem to have afforded contributive elements to facilitate deflection of the ball downward and forward.

I obtained a cadaver six feet high, but the body not quite as stout as that of the President, suspended it so that the feet rested on the floor, making it assume as near as possible the position in which the President stood when shot. A twelve inch trochar (a steel rod one-quarter inch in diameter, pointed at the end and fixed in a handle), was entered exactly at the point of the President's wound and made to pin all the tissues and organs together, so as to steady them during the progress of dissection. A careful dissection of the right loin was then made, layer by layer, from the skin to the internal organs, from an area extending from the tenth rib above to the crest of the ilium below, and from the median line flaps of the layers were turned off to the side. In the track of the wound the skin, sub-cutaneous tissue, the latissimus dorsi muscle plane and that of the serratus posticus inferior muscle were removed, exposing the tenth, eleventh and twelfth ribs and their intervening muscles; also the exterior surface of the transversalis muscle for the external half of the loin and the erector spinæ muscle for the inner half of the loin. The external intercostal muscles were dissected out of the intercostal spaces and the intercostal vessels and nerves recognized, as also the costal layer of the pleura bridging the inner portion of the spaces to line the interior surfaces of the rib; in the external portion of the intercostal spaces; after the removal of the external intercostals, the posterior position of the internal intercostal plane of muscle was presented. The costal layer of the pleura was separated from the eleventh rib and one inch of the rib corresponding to the probable point of fracture was cut out with bone forceps. The pleural cavity was opened, and, the finger being inserted, found a pocket extending down to the twelfth rib, as the pleural attachment rises posteriorly toward the spine and anteriorly toward the eleventh rib. Three planes of tissue covering the convexity of the liver were now brought into view and were presented in the following order:—First, the diaphragmatic layer of the pleura; second, dissecting this off exposed the muscle structure of the diaphragm (the diaphragm being attached to the eleventh and twelfth ribs); third, the peritoneal lining of the abdominal surface of the diaphragm. Cutting through the peritoneum brought into view the thick lateral superior border of the right lobe of the liver. Removing the diaphragm a little lower down there was exposed the external and upper border of the kidney, there being no peritoneum intervening.

A large director was slipped in anterior to the tip of the twelfth rib in a direction downward on to the internal surface of the transversalis muscle. It passed readily downward and inward, following the course of the loin into the iliac fossa. The twelfth rib was then cut through and dissected out with the bone forceps, carrying away its diaphragmatic attachment; the transversalis muscle was sectioned along the director, down to the crest of the ilium. The ilio-hypogastric and ilio-inguinal nerves were dissected out and preserved *in situ.* The hand was inserted, crowding the fat behind the kidney, with the large intestine (the peritoneal sac being intact) forward; the fingers passed with great facility downward and inward into the iliac fossa until the palmar surface of their tips rested on the iliacus internus muscle, the nails against the external and inferior surface of the psoas magnus muscle (this particular point is situated at the back and internal bed of the groin.) The fingers, when crowded a little, passed to the surface of the anterior crural and obturator nerves of the lumbar plexus; crowding them still further the lumbo-sacral cord of the sacral plexus and even the sacral plexus itself could be felt. The peritoneum was now opened anterior to the external convex border of the kidney, exposing the external lateral border of the right lobe of the liver. The fat from the kidney was removed, and the organ was recognized as bedded in the fossa in the posterior and inferior surfaces of the right lobe of the liver. A ball to reach the external border of the right lobe of the liver would have to enter the cavity of the peritoneum and then penetrate the liver.

The dissection being now completed and the relation of the organ involved in the region of the liver being rendered appreciable, a careful drawing, by Mr. Max Cohn, of the region was made, which, upon subsequent comparison, was found to accord exactly with the illustration of the region made by our standard authorities. The dissection of the region thus seemed to give additional possibility to the theory of the course of the ball and its present lodgment.

On the morning of Thursday, July 7, I called upon Professor Frank H. Hamilton, M.D., and stated to him my theory of the probable deflection and course of the ball, giving him my reasons therefor and my investigations and the data I had obtained relative to the bullet and the pistol and the results of my dissections of the injured region. I then asked the doctor to give me a detailed statement of all the facts in the case that he had learned at the time of his visit to Washington as one of the consulting surgeons. This he kindly gave me, and among other symptoms stated that the President had called attention to a peculiar sensitiveness of the skin of the right side of the scrotum. An injury to the ilio-hypogastric and ilio-inguinal nerves, which lie in the supposed track of the ball, according to the theory advanced would account for this peculiar sensation. This, indeed. seemed to be a most happy confirmation of the probable correctness of the theory. Encouraged by Professor Hamilton I repeated my dissections on July 7, and he made an appointment to be present at a demonstration of the same on the following day. On July 8 I obtained several cadavers resembling the physique of the President, and at two P. M., in the presence of Dr. Hamilton, Dr. George F. Shrady, editor of the New York *Medical Record*, and other professional gentlemen, I repeated the dissections, pistol firings, etc. I propose to continue these dissections, and carry them still further, in order, if possible, to determine the probabilities as to how eventually the ball will be dislodged and where it will most likely point, if, perchance, it is where I think it likely to be. FANEUIL D. WEISSE,

*Professor of Practical and Surgical Anatomy in the Medical Department of the University Medical College of New York.*

The Professor having telegraphed July 8, to the Union Metallic Cartridge Company, Bridgeport, Conn., for data, with reference to the 41 cartridge, received in answer the following telegram:

UNION METALLIC CARTRIDGE COMPANY,
BRIDGEPORT, CONN., July 9, 1881.

In reference to the 44 central fire cartridge, would say that the exact amount of powder contained in the cartridge is twenty grains; weight of bullet 200 grains. The crimping or turning in of the shell in the end makes the cartridge shoot stronger. C. L. RICHMOND.

The *Herald* also contains an interview with Prof. Frank H. Hamilton, who has been at Washington in consultation on the President's case, and who expressed the opinion that there is no conclusive proof of a wound of the liver.

Dr. Weisse makes it highly probable that the bullet glanced downward behind the liver without touching it, but the further course of it as marked out in his theory seems very improbable. That the shot should enter the abdomen through the eleventh rib, slide down to the pelvis, and curve across the iliac fossa without wounding the colon, kidney, ureter, anterior crural nerve, nor either of the iliac arteries, and then, turning abruptly backward into the hollow of the sacrum, wound neither the bladder nor rectum, but injure there the roots of both the right and left sciatic nerves, so as to cause the pain in both feet and legs, seems, though not absolutely impossible, very difficult to believe. The supposed lodgment in the hollow of the sacrum without wounding arteries or viscera, and the injuring the sacral nerves so as to cause pain without paralyzing motion is highly improbable, for the motor and sensory fibers there run mingled in the same trunks, and would be alike injured. It is more probable that when the eleventh rib was shattered the corresponding costal nerve received a violent jerk, which affected the adjacent cord mechanically where the sensory tract is sufficiently separate from the motor columns to receive an isolated injury. This would account for the sensory disturbance of the feet and legs better than a wound of the sacral plexus. If the liver has been really wounded it would not necessarily be fatal. The statistics of the late war, published by the War Department, may be condensed as follows:

Uncomplicated gunshot wounds of the liver—Cases, 59; deaths, 34; mortality 58 per cent.

Gunshot wounds of liver, complicated with other injuries—Cases, 114; deaths, 74; mortality, 66 per cent.

The army statistics have very little value for determining the President's risk, because the correct diagnosis was frequently impossible, and the projectile used in the regulation musket was much larger than the one fired by Guiteau, still they show that even if the liver is really wounded it will not necessarily be a fatal case. As above stated, I consider the present location of the projectile entirely undetermined, but that the probabilities are against it being either in the liver, or in the hollow of the sacrum.

No. 6, Sixteenth Street, Chicago.

